# Past incarceration and chlamydia infection among young Black men in New Orleans

**DOI:** 10.3389/fpubh.2023.1114877

**Published:** 2023-03-27

**Authors:** Jenisha L. Stapleton, Aneeka Ratnayake, Gérard Gomes, Hua He, Patricia J. Kissinger

**Affiliations:** Department of Epidemiology, Tulane University School of Public Health and Tropical Medicine, New Orleans, LA, United States

**Keywords:** jail, prison, incarceration, young Black men, Deep South, post-release

## Abstract

**Background:**

Young Black men are disproportionately and adversely affected by incarceration and sexually transmitted infections (STIs), both of which share common social and structural determinants. It is well documented that incarcerated individuals, including youth, are more likely to acquire STIs in the carceral setting compared to the general population. However, the effects of imprisonment on sexual health outcomes after imprisonment are not well-understood. The relationship between incarceration history (having ever spent time in a correctional institution such as prison, jail, or juvenile detention) and chlamydia positivity was examined in this study.

**Methods:**

A secondary analysis of the *Check it* Program, a *Chlamydia trachomatis* (Ct) community-based seek, test, and treat screening program for Black men aged 15–24 who have sex with women in New Orleans was conducted. Participants completed a computer-assisted self-administered questionnaire on relevant sexual and social histories and provided a urine specimen for a Ct urine nucleic acid amplification test. Bivariate and multivariable regressions were used to estimate the association between incarceration history and chlamydia positivity.

**Results:**

Participants (*N* = 1,907) were enrolled from May 2017 to March 2020. Of those, 351/1,816 (19.3%) reported past incarceration and 203/1,888 (10.8%) tested positive for Ct. When adjusted for age, insurance status, and condom use, having a history of incarceration was positively associated with a positive Ct test (adjusted odds ratio (95% confidence interval):1.61 (1.12, 2.31), *p* = 0.0095).

**Conclusions:**

Interacting with the carceral system is associated with a positive Ct test post-incarceration. Incarceration may be an important marker for Ct acquisition in young Black men who have sex with women and those with a history of incarceration should be prioritized for Ct screening after release.

## Introduction

Caused by the *Chlamydia trachomatis* (Ct) bacterium, Ct accounts for the largest proportion of reportable STIs in the United States (US) with 1.6 million cases reported in 2020 (1). Untreated Ct infections in men can lead to proctitis, urethritis, and infertility and can serve as a reservoir of infection in women (2). In 2018, Ct cases among Black men were 6.8 times the rate compared to White men (952.3 and 140.4 cases per 100,000 population, respectively) (3). Among males aged 15–19 years, reported Ct cases among Black men were 9.1 times the rate among White men (2,668.6 and 293.0 cases per 100,000 population, respectively). Reported Ct cases among Black males aged 20–24 years were 5.3 times the rate among White males of the same age group (3,867.1 and 732.6 cases per 100,000 population, respectively) (3).

In Louisiana, a state in the Deep South, the Ct diagnosis rate was 777.2 cases per 100,000 population with a 5% increase in males from 11,068 cases in 2018 to 11,599 cases in 2019 (4). In the same year, 70% of all Ct cases with reported race were Black individuals, a rate that demonstrates a significant racial disparity as Black people comprise only 32% of Louisiana's population (5). Additionally, 69% of all cases were among youth younger than 25 years old, with the highest rates among youth aged 15–19 and 20–24 (4). Even after adjusting for individual-level factors that impact risk, such as the number of sexual partners or condomless sex, disparities in Ct outcomes still remain. This suggests that other community- or structural-level factors may play a role in the observed disparities.

Structural racism is an important social determinant of population health in the United States (6, 7) as racial minorities bear a disproportionate burden of general morbidity (8). Literature on the sexual risk behaviors of youth of color describes several pathways through which racism leads to unhealthy conditions. Consequently, these unhealthy conditions can facilitate the observed elevated risks of STIs in youth of color (9). Boutrin and Williams organize the three key mechanisms of racism into institutional/structural racism, cultural racism, and differential treatment (discrimination) by both institutions and individuals (9). Structural racism can affect stress and behaviors through residential segregation, criminal justice system policies, and incarceration (9).

It is well documented that those incarcerated are more likely to acquire STIs in the carceral setting compared to the general population due to drug use, high-risk sexual behaviors, densely populated settings, and reduced access to screening (10). In a systematic review of STI prevalence among prisoners, the prevalence of STIs among prisoners was higher than the general population with drug use, low educational levels, and unsafe sexual activities as major risk factors (11). However, it is not well understood if interacting with the carceral system influences post-incarceration Ct rates.

Mass incarceration is an institutional driver of racial inequality (12). The US has the largest prison population and the highest rates of imprisonment and incarceration in the world (13). In 2021, Louisiana had the highest incarceration rate in the US, with 1,094 people per 100,000 population in state prisons, local jails, federal systems, and other systems of confinement (14). By race, Black people are overrepresented in prisons and jails in Louisiana with 1,411 incarcerated Black people per 100,000 Black people in the state in 2019. This is a 3.8 to 1 disparity ratio to White people (15). As of June 2021, 95.4% of the prison population were men, 6,490 individuals were admitted between the ages of 18 and 24, and 1,182 inmates of the same age were released the same year (16).

Research examining the relationship between incarceration rates and STI rates and incidence has been scarce. High rates of STIs often occur in communities where incarceration rates are also high. Initial cross-sectional county-level studies have found positive correlations between incarceration rates and STI rates in the general population (17–19). These studies examined incarceration in men and women collectively and demonstrated that the relationship was strongest with correlating STIs occurring in the year after the incarcerations. In a longitudinal analysis using census tract-level data of male incarceration rates in a south-eastern city, census tracts with higher baseline male incarceration rates had higher baseline rates of incident STIs (20). Among men in the US, incarceration has also been associated with high-risk sex partnerships, such as multiple partners and condomless sex, which are known risk factors for STIs (21). In another study of incarceration and risky sexual behavior, men reporting incarceration in the past 12 months were 1.8 times as likely to experience multiple new sexual partnerships and transactional sex in the past 4 weeks (22). However, the effects of imprisonment on sexual health outcomes after imprisonment are not well-understood.

In a retrospective cohort study of having a positive Ct, gonorrhea, or syphilis test or incident-positive HIV test in the year following arrest or incarceration, test positivity rates were highest for chlamydia and rates of positive STIs and HIV were between 2.7 and 6.9 times higher for Black people than White people with an arrest or incarceration between 2003 and 2008. Compared to those without an arrest or incarceration, those with arrest or incarceration had a relative risk of 3.9 for Ct (23). Furthermore, Black race for men and younger age for Ct and gonorrhea were among the characteristics that were most associated with increased risk for positive STI in a cohort of individuals released from jail (24). These few existing studies motivated this present study since incarceration history and STI outcomes have not been studied specifically among young Black men.

Given the high and ever-increasing incarceration rates in the US and high rates of STIs, which disproportionately affect young Black men in the Deep South, incarceration may be an important social determinant of Ct risk in young Black men. At the intersection of race-based incarceration and disparities in sexually transmitted infections, this study investigates Ct outcomes in young Black men with and without exposure to prior incarceration. The relationship between incarceration history (having ever spent time in a correctional institution such as prison, jail, or a juvenile detention center) and Ct positivity in New Orleans, Louisiana was examined in this study. It is hypothesized that Ct positivity will be greater in those with a history of incarceration.

## Methods

The *Check It* Program was a *Chlamydia trachomatis* (Ct) community-based seek, test, and treat screening program for Black men aged 15–24 who have sex with women in New Orleans, Louisiana. Methods have been described elsewhere, but briefly, the program was developed to evaluate the effect of screening and treating men for chlamydia and gonorrhea in communities with high STI prevalence (25). Participants were recruited through venue-based recruitment and via print and social media marketing. Venues included barbershops, historically Black colleges or universities, Black-owned businesses, or other locations where young Black men frequented. At physical venues, field staff approached participants about the study. Eligible participants provided informed consent, completed a computer-assisted self-administered survey that elicited relevant sexual and social histories, provided a urine sample, and received a wide variety of monetary or in-kind compensation. In-kind compensation consisted of vouchers for services or gift cards for community partners and venues valued at $25. This study was approved by Tulane University's Institutional Review Board.

Participation in *Check It* was limited to those who met the following inclusion criteria: identified as African American or Black, were assigned male sex at birth (based on the question “do you have a penis?”), were 15–24 years of age, had lived or spent most of their time in Orleans Parish, had reported having had vaginal sex with a woman in their lifetime, were able and willing to consent to study activities, spoke and understood English, had not taken azithromycin in the 2 weeks before enrollment, and had not been previously enrolled in *Check It* (25).

This analysis was restricted to participants enrolled from the beginning of the study in May 2017 until March 2020, when the COVID-19 stay-at-home mandate was implemented in New Orleans. After restricting to account for the impact of this secular trend on sexual behavior and consequently Ct infections, the final dataset had 1,907 young men. Of those participants, 94.9% (1,809/1,907) had both complete information on incarceration history and conclusive chlamydia screening results. After removing participants with missing covariate data, the sample size for the primary complete case analysis was 1,616 participants. To assess the impact of missing data, multiple imputation was conducted in a sensitivity analysis to address the missing data.

Respondents were asked about their experiences with the criminal justice system. The primary exposure of ever having spent time in jail was reported retrospectively when respondents were asked, “Have you ever spent time in a correctional institution such as a prison, jail, or juvenile detention?” Response options included “yes”, “no”, or “refuse to answer”. The survey instrument only captured data on lifetime incarceration (ever incarcerated) and did not capture data on when the incarceration occurred, such as in the last 6 months. Cases in which respondents selected “refuse to answer” were recoded as missing.

The outcome measure of chlamydia was assessed using a nucleic acid amplification test (NAAT) from a first-catch urine sample from the participant. Results of the screening were categorized as “negative”, “positive”, or “specimen error”. Respondents whose samples resulted in a specimen error were recoded as missing.

Age was constructed as a continuous variable calculated based on the date of birth and the date of enrollment and then categorized into two groups (15–18 and 19–24). Dichotomous variables were also constructed for insurance status, condom use, and binge drinking. For the age at sexual onset, participants were asked, “How old were you the first time you had any kind of sex? That includes vaginal, oral, or anal sex.” and responses ranged from < 14 years old to 25 years (categorized as ≤ 14–25 in one-unit increments). Participants were asked about the number of female sexual partners in the previous 2 months and lifetime male and female partners and responded with a whole number.

Descriptive analysis was performed to describe the prevalence of incarceration, chlamydia, and other characteristics of the entire cohort. The median and range were provided for continuous variables and a count and percentage were provided for categorical variables. The cohort characteristics were stratified by the participants' incarceration history and chlamydia test result for bivariate analyses. Inferential statistics (*p*-values) were obtained using Mann-Whitney U tests for continuous variables and Pearson Chi-Squared tests for categorical variables. Descriptive and bivariate analyses were conducted using IBM SPSS Statistics Version 28.0. (26).

In the primary analysis, bivariate and multivariable logistic regressions were used to estimate the crude and adjusted associations (odds ratios, 95% confidence intervals, and *p*-values) between history of incarceration and chlamydia positivity. Respondents who selected “refuse to answer” were recoded as missing. Observations with missing values for the outcome, exposure, or covariate data were not included in the complete case primary analysis. Covariates associated with both incarceration and chlamydia at a two-tailed significance level of 0.05 or less were tested as potential confounders. These variables were categorical age, insurance status, age at sexual debut, condom use, binge drinking, number of lifetime female sexual partners, and parental incarceration. Forward, backward, and stepwise selections were used to determine which covariates to include in the final model. The significance level for entering or staying in the model was 0.05. Categorical age, insurance status, and condom use were consistently selected across all three selection methods and were retained for the final adjusted model. The Hosmer-and-Lemeshow goodness of fit test was used to assess model fit. Bivariate and multivariable logistic regressions were performed using SAS Version 9.4. (27).

A sensitivity analysis was conducted to assess the impact of missing values. From examining missingness among the variables of interest, age at sexual debut, the number of lifetime female sexual partners, and condom use were identified as auxiliary variables. They were correlated (correlation coefficient < 0.4) with the participants' incarceration history. Mean comparisons via statistical testing revealed that the total number of female sexual partners were associated with the missingness of participants' incarceration history and chlamydia test results. The mean number of lifetime female sexual partners was significantly higher among respondents who had missing data on incarceration history (*p* < 0.001) and chlamydia test results (*p* = 0.0139). The mean age at sexual debut was significantly higher among respondents who had missing data on incarceration history (*p* < 0.001). Condom use was also associated with missingness of participants' incarceration history (*p* = 0.0111). As such, these variables were included in the imputation model to address missing values. The fully conditional specification method was used to impute missing values. Dichotomous variables were imputed under a logistic distribution and continuous variables were imputed using the predictive mean matching method. Fifteen imputed datasets were created. Binary logistic regression analyses were conducted on the imputed datasets and those results were synthesized for inference using SAS Version 9.4. (27). **Table 5** reports crude and adjusted associations (odds ratios, 95% confidence intervals, and *p*-values) obtained using the multiply imputed datasets with missingness in the data addressed, for reference (27).

## Results

Table 1 presents the descriptive statistics of the participants (*N* = 1,907) enrolled through March 2020. The median age was 19.8 years (range of 9.9), and all participants identified as Black/African American with 3.3% (62/1897) identifying as two or more races, and 3.1% (58/1897) identifying as Hispanic. Based on the analytical sample, 60.6% (1156/1885) were high school students or graduates and 38.2% (729/1885) had higher than a high school education. Having a form of health insurance was reported by 80.3% (1442/1795) of participants. Money or services from government assistance programs were received by 35.3% (674/1907) of the participants or a member of their household. About 19% (351/1816) of the participants reported having spent time in prison, jail, or a juvenile detention center. Previous chlamydia (Ct) test results were reported for 40.6% (762/1879) of participants and 10.8% (203/1888) had positive Ct test results during the study.

**Table 1:** Characteristics of the *Check It* cohort enrolled through March 2020 (*n* = 1,907).

	**Sample size**	**Participants enrolled through March 2020 (*n =* 1,907)**
**Age (median years, range)**	1,899	19.8 (9.9)
**Categorical age**		
15–18		722 (38%)
19–24		1,177 (62%)
**Race**	1,897	
Black/African American only		1,835 (96.7%)
Two or more races		62 (3.3%)
**Ethnicity**	1,897	
Hispanic		58 (3.1%)
Non-Hispanic		1,835 (96.7%)
**Education**	1,885	
High school graduate or less		1,156 (60.6%)
More than high school		729 (38.2%)
**Insurance**	1,795	
No insurance		353 (19.7%)
Some type of insurance		1,442 (80.3%)
**Participant or household used government assistance in the past 12 months**	1,907	
Yes		674 (35.3%)
No		1,233 (64.7%)
**Binge drinking in the past 2 months**	1,369	
At least once		512 (27.2%)
No		1,369 (72.8%)
**Any drug use in the past 2 months**	1,849	
At least one type of drug		1,120 (60.6%)
No		729 (39.4%)
**Only marijuana used in the past 2 months**	1,860	
Only marijuana		975 (52.4%)
No drugs or drugs other than marijuana		885 (47.6%)
**Age at sexual debut (median years, range)**	1,855	15.0 (11)
**Consistent condom use with all partners in past 2 months**	1,723	
Yes		741 (43.0%)
No		982 (57.0%)
**Sexual partners in the past 2 months**	1,869	
Only one female partner		1,207 (64.6%)
Two or more female partners		662 (35.4%)
**Total female sexual partners in lifetime (median, range)**	1,855	5 (90)
**Male partners in lifetime**	1,828	
At least one male partner		97 (5.3%)
No male partners		1,731 (94.7%)
**Own incarceration**	1,816	
Yes		351 (19.3%)
No		1,465 (80.7%)
**Ever booked or charged**	1,821	
Yes		373 (20.5%)
No		1,448 (79.5%)
**Ever convicted (not minor traffic)**	1,820	
Yes		211 (11.6%)
No		1,609 (88.4%)
**Previous chlamydia test**	1,879	
Yes		762 (40.6%)
No		1,117 (59.4%)
**Chlamydia test results**	1,888	
Positive		203 (10.8%)
Negative		1,685 (89.2%)

In the 2 months prior to enrollment, binge drinking was reported by 27.2% (512/1369) of the participants, 60.6% (1120/1849) used at least one type of drug, and 52.4% (975/1860) used marijuana only (87.1%, 975/1120 of those using drugs). The median age of sexual debut was 15.0 (range of 11). Nearly half of the participants (43.0%, 741/1723) used condoms consistently with all female partners, 64.6% (1207/1869) of participants had one female partner only, and 35.4% (662/1869) had two or more female partners 2 months prior to enrollment. The median number of lifetime female partners was 5 (range of 90). A few participants (5.3%, 97/1828) reported at least one male partner in their lifetime.

Table 2 presents the distribution of demographic and behavioral characteristics by the participants' own history of incarceration. Time spent in jail was reported by 19.3% (351/1816) participants and was associated with older age (*p* < 0.001), having no health insurance (*p* < 0.004), use of government assistance (*p* < 0.001), younger age at sexual debut (*p* < 0.001), inconsistent condom use (*p* < 0.001), binge drinking (*p* = 0.009), at least one type of drug use (*p* < 0.001), marijuana use (*p* < 0.001), and more lifetime female sexual partners (*p* < 0.001), parental incarceration (*p* < 0.001), previous Ct tests (*p* < 0.001), and positive Ct test results (*p* = 0.002). Participants' own history of incarceration was not associated with the number of male sexual partners in the prior 2 months or lifetime.

**Table 2:** Characteristics by participants' incarceration history (*n* = 1,816).

**Characteristic**	**Spent time in jail (*n =* 351) *n* (%)**	**No time spent in jail (*n =* 1,465) *n* (%)**	***p*-value**
**Age (median years, range) (*****n** =* **1,816)**	20.6 (10.0)	19.7 (10.0)	**< 0.001** ^**a**^
**Categorical age**			
15–18	113 (32.2)	572 (39.0)	**0.017^a^**
19–24	238 (67.8)	893 (61.0)	
**Race (*****n** =* **1,816)**			
Black/African American only	340 (98.9)	1,417 (96.7)	0.892^a^
Two or more races	11 (3.1)	37 (3.1)	
**Ethnicity (*****n** =* **1,814)**			
Hispanic	12 (3.4)	44 (3.0)	0.689^a^
Non-Hispanic	339 (96.6)	1,419 (97.0)	
**Education (*****n** =* **1,809)**			
High school graduate or less	243 (69.6)	864 (59.2)	**< 0.001^a^**
More than high school	106 (30.4)	243 (40.8)	
**Insurance (*****n** =* **1,724)**			
No insurance	84 (24.9)	251 (18.1)	**0.004^a^**
Some or other type of insurance	253 (75.1)	1,136 (81.9)	
**Participant or household used government assistance in the past 12 months (*****n** =* **1,816)**			
Yes	152 (43.3)	501 (34.2)	**< 0.001^a^**
No	199 (56.7)	964 (65.8)	
**Age at sexual debut (median years, range)** ***n** =* **1,798**	14 (11.0)	15 (11.0)	**< 0.001^b^**
**Consistent condom use (*****n** =* **1,699)**			
Yes	106 (32.1)	631 (46.1)	**< 0.001^a^**
No	1,076 (73.8)	738 (53.9)	
**Binge drinking in the past 2 months (*****n** =* **1,808)**			
No	234 (66.9)	1,076 (73.8)	**0.009^a^**
At least once	116 (33.1)	382 (26.2)	
**Any drug use in the past 2 months (*****n** =* **1,785)**			
No	91 (26.1)	619 (43.1)	**< 0.001^a^**
At least one type of drug	257 (73.9)	818 (56.9)	
**Only marijuana used in the past 2 months (*****n** =* **1,785)**			
No drugs or drugs other than marijuana	137 (39.4)	706 (49.1)	**0.001^a^**
Only marijuana	211 (60.6)	731 (50.9)	
**Sexual partners in the past 2 months (*****n** =* **1,789)**			
Only one female partner	212 (61.1)	952 (66.0)	0.084^a^
Two or more female partners	135 (38.9)	490 (34.0)	
Total female sexual partners in lifetime (median, range) *n =* 1,747	7.0 (90.0)	5.0 (90.0)	**< 0.001^b^**
At least one male partner in lifetime (*n =* 1,775)	17 (4.9)	75 (5.2)	0.822^a^
No male partners in lifetime	327 (95.1)	1,356 (94.8)	
**Previous chlamydia test (*****n** =* **1,808)**			
Yes	179 (51.1)	546 (37.4)	**< 0.001^a^**
No	171 (48.9)	912 (62.6)	
**Chlamydia test results (*****n** =* **1,809)**			
Positive	54 (15.4)	141 (9.7)	**0.002^a^**
Negative	296 (84.6)	1,318 (90.3)	

a Pearson Chi-Square Test.

b Mann-Whitney U-Test.

Bold values represent statistical significance at or below *p* = 0.05.

Table 3 presents the bivariate analysis of demographic and behavioral characteristics by Ct positivity. About a tenth of the participants (10.8%, 203/1888) tested positive for Ct, and positive results were associated with older age (*p* = 0.002), having no health insurance (*p* = 0.011), the participant's own history of incarceration (*p* = 0.002), younger age at sexual debut (*p* = 0.016), inconsistent condom use (*p* = 0.004), binge drinking (*p* = 0.006), having two or more female sexual partners in a lifetime (*p* < 0.001), and more lifetime female sexual partners (*p* < 0.001). Positive Ct test results were not associated with the use of government assistance, any drug use, marijuana use, the number of male sexual partners, and previous Ct screening.

**Table 3:** Characteristics by chlamydia test results (*n* = 1,888).

	**Positive test (*n =* 203) *n* (%)**	**Negative test (*n =* 1,685) *n* (%)**	***p*-value**
**Age (median years, range)**	20.4 (9.9)	19.7 (9.9)	**0.002^b^**
**Categorical age**			
15–18	57 (28.1)	656 (39.0)	**0.002** **^a^**
19–24	146 (71.9)	1,024 (61.0)	
**Race (*****n** =* **1,883)**			
Black/African American only	200 (98.5)	1,621 (96.5)	0.125^a^
Two or more races	3 (1.5)	59 (3.5)	
**Ethnicity (*****n** =* **1,804)**			
Hispanic	6 (3.0)	51 (3.0)	0.956^a^
Non-Hispanic	196 (97.0)	1,626 (97.0)	
**Education (*****n** =* **1,871)**			
High school graduate or less	108 (53.7)	1,039 (62.2)	**0.020^a^**
More than high school	93 (46.3)	631 (37.8)	
**Insurance (*****n** =* **1,782)**			
No insurance	50 (26.6)	300 (18.8)	**0.011^a^**
Some or other type of insurance	138 (73.4)	1,294 (81.2)	
**Own incarceration (*****n** =* **1,809)**			
Yes	54 (27.7)	296 (18.3)	**0.002^a^**
No	141 (72.3)	1,318 (81.7)	
**Participant or household used government assistance in the past 12 months (*****n** =* **1,888)**			
Yes	63 (31.0)	605 (35.9)	0.170^a^
No	140 (69.0)	1,080 (64.1)	
**Age at sexual debut (median years, range) (*****n** =* **1,843)**	14 (11)	15 (11)	**0.016^b^**
**Consistent condom use (*****n** =* **1,713)**			
Yes	62 (33.2)	676 (44.3)	**0.004^a^**
No	125 (66.8)	850 (55.7)	
**Binge drinking in the past 2 months (*****n** =* **1,868)**			
No	129 (64.5)	1,230 (73.7)	**0.006^a^**
At least once	71 (35.5)	438 (26.3)	
**Any drug use in the past 2 months (*****n** =* **1,837)**			
No	70 (35.2)	653 (39.9)	0.201^a^
At least one type of drug	129 (64.8)	985 (60.1)	
**Only marijuana used in the past 2 months (*****n** =* **1,842)**			
No drugs or drugs other than marijuana	86 (43.2%)	784 (47.7%)	0.230^a^
Only marijuana	113 (56.8%)	859 (52.3%)	
**Sexual partners in the past 2 months (*****n** =* **1,856)**			
Only one female partner	110 (54.2%)	1,092 (66.1%)	**< 0.001^a^**
Two or more female partners in lifetime	93 (45.8%)	561 (33.9%)	
Total female sexual partners in lifetime (median, range) (*n =* 1,783)	8 (89)	5 (90)	**< 0.001^b^**
At least one male partner in lifetime (*n =* 1,819)	9 (4.7%)	87 (5.4%)	0.686^a^
No male partners in lifetime	184 (95.3%)	1,539 (94.6%)	
**Previous chlamydia test (*****n** =* **1,866)**			
Yes	82 (41.0%)	673 (40.4%)	0.869^a^
No	118 (59.0%)	993 (59.6%)	

a Pearson Chi-Square Test.

b Mann-Whitney U-Test.

Bold values represent statistical significance at or below *p* = 0.05.

Table 4 presents the bivariate and multivariable logistic regression analyses for the participants' own history of incarceration and Ct positivity. There was a positive crude association between having a history of incarceration and testing positive for Ct using the primary complete case analysis [OR (95% CI): 1.71 (1.22, 2.39), *p* < 0.0020)]. In the multivariable logistic regression model, categorical age, insurance status, age at sexual debut, consistent condom use, binge drinking, number of lifetime female sexual partners, and parental incarceration were examined for potential confounding effects. Categorical age, insurance status, and condom use were consistently selected across all three selection methods and were retained for the final adjusted model. The final adjusted model [aOR (95% CI): 1.61 (1.12, 2.31), *p* = 0.0095] was assessed for the goodness of fit and the results of the Hosmer-and-Lemeshow goodness-of-fit test supported that the model fitted the data well (*p* = 0.4254). The results of the sensitivity analysis, as seen in Table 5, were similar to the results based on the primary analysis. As such, the primary results remain robust.

**Table 4:** Crude and adjusted associations for chlamydia outcomes and history of incarceration from primary complete case analysis.

	**Crude model OR (95% CI)**	***p*-value**	**Adjusted model^a^ OR (95% CI)**	***p*-value**
**History of incarceration**				
No	Reference		Reference	
Yes	1.71 (1.22, 2.39)	0.0020	1.61 (1.12, 2.31)	0.0095
**Consistent condom use**				
Yes	Reference		Reference	
No	1.60 (1.16, 2.21)	0.0038	1.43 (1.02, 2.01)	0.0374
**Categorical age**				
15–18	Reference		Reference	
19–24	1.64 (1.19, 2.26)	0.0025	1.47 (1.02, 2.10)	0.0383
**Insurance**				
Some or other type of insurance	Reference		Reference	
No insurance	1.56 (1.11, 2.21)	0.0117	1.46 (1.01, 2.10)	0.0450

a Adjusted for categorical age, insurance status, and condom use.

**Table 5:** Crude and adjusted associations for chlamydia outcomes and history of incarceration from sensitivity analysis.

	**Crude model OR (95% CI)**	***p*-value**	**Adjusted model^a^ OR (95% CI)**	***p*-value**
**History of incarceration**				
No	Reference		Reference	
Yes	1.66 (1.52,1.80)	< 0.0001	1.51 (1.38, 1.65)	< 0.0001
**Consistent condom use**				
Yes	Reference		Reference	
No	1.59 (1.47,1.72)	< 0.0001	1.42 (1.31, 1.54)	< 0.0001
**Categorical age**				
15–18	Reference		Reference	
19–24	1.65 (1.52,1.79)	< 0.0001	1.48 (1.36, 1.61)	< 0.0001
**Insurance**				
Some or other type of insurance	Reference		Reference	
No insurance	1.55 (1.42, 1.69)	< 0.0001	1.44 (1.32, 1.57)	< 0.0001

a Adjusted for categorical age, insurance status, and condom use.

## Discussion

In this study, incarceration history was associated with chlamydia (Ct) positivity in young Black men who have sex with women in New Orleans, as hypothesized. Those who had a history of incarceration had a 61% increased risk in testing positive for Ct compared to those who did not have a history of incarceration, after accounting for the effects of age, insurance status, and condom use. The positive trend for crude and adjusted associations remained with the more stringent sensitivity analysis and supports the conclusions of the primary analysis. These findings demonstrate that past incarceration is associated with Ct risk in young Black men. It also supports that incarceration is an important social determinant of sexual health outcomes that negatively impact vulnerable populations including young Black men.

Incarceration can have devastating effects on sexual networks of young Black men by modifying their composition in a manner that can contribute and even accelerate the spread of STIs (28). Removing men from a community can disrupt existing sexual relationships during and after incarceration (18, 29, 30). During incarceration men may engage in same-sex sexual behavior, such as condomless anal sex, which increases the risk of STI transmission (18, 28). Moreover, recently released men may return to previous sexual relationships without disclosing sexual activities during incarceration and may also form new concurrent sexual relationships (18, 31). These factors may help to explain the mechanism by which male incarceration may contribute to increased STI incidence and prevalence in the population after release from incarceration.

There were strengths to this study. Firstly, it allowed us to examine a minoritized subgroup of the Black population. Where previous studies have stratified analyses by race, this study elicited within race differences among a group of young Black men. Furthermore, this was as a relatively large sample of young Black men. Additionally, participants' urine was tested for Ct during the study; thus, avoiding reporting bias, which STIs has been known to be under-reported in minorities who experience additional stigma (32).

There were also some limitations that must be considered. Incarceration was self-reported and those who did not report a history of incarceration may have been subject to social desirability bias since incarceration is highly stigmatized. Arrest records were not accessed to verify incarceration history; however, there is no reason to suspect recall bias in participants that reported a history of incarceration because recalling traumatic or adverse experiences such as their own incarceration has reliable estimates (33). As with all cross-sectional data, establishing a temporal relationship is challenging because the data does not allow us to discriminate between infection acquisition. That is, it is unknown whether the infection was acquired during incarceration and persisted post-release or was acquired during post-release. This is important to note as Ct infection is generally asymptomatic and can be harbored for a long time. We also do not know how long ago the incarceration occurred. It is possible that incarceration is a proxy for all the other factors that were more common among the formerly incarcerated group, such as having no health insurance, use of government assistance, younger age at sexual debut, and parental incarceration. However, many factors known to be associated with both incarceration and Ct positivity were measured and adjusted for, minimizing the likelihood that the observed results were due to a proxy related to incarceration. Additionally, the study may be vulnerable to sampling bias because the sample was a convenience sample of sexually active young Black men. As such, it may not be generalizable to the target population of young Black men in New Orleans. Since data on community-level characteristics (such as neighborhood segregation and social capital) were not collected, it is possible that residual confounding may have remained after adjusting for confounding in the analysis.

The effects of age at incarceration, duration of incarceration, multiple incarcerations (recidivism) and duration of time since release were not captured in the *Check It* survey and should be investigated in future studies that include longitudinal analysis. Duration of incarceration and time since release from incarceration may provide nuanced insights and help to identify a critical period of heightened risk for incident STI cases. This can inform prevention and intervention programs that target formerly incarcerated young men as the duration of incarceration and the one-year period from incarceration represent a high-impact opportunity to reduce STIs (23, 34, 35). Furthermore, the exposure measure could be disaggregated (jail, prison, and juvenile detention separately) to discern the relationship between the various carceral settings as the relationship between STIs and incarceration have been shown to be stronger with prison than jail incarceration (18). Certain community-level variables could not be obtained such as gender-ratio imbalances, which are linked to the number of opposite-sex sexual partners post-release and could be an important covariate for future work in understanding correlates of STI burden in young Black men (36, 37).

The present study contributes to emerging evidence that incarceration may function as a driver for Ct incidence and prevalence. It strengthens support that mass incarceration, perpetuated by structural racism, has negative consequences for sexual health outcomes in formerly incarcerated young Black men, a group already marginalized, in a southern US city. Establishing criminal justice policies that prioritize community re-entry for formerly incarcerated individuals, promoting community health services that focus on screening individuals recently released from carceral systems, and preventing criminal justice involvement can mitigate these unintended negative effects of poor sexual health outcomes in the southern US, which is burdened by high rates of incarceration and STIs in young Black men.

## Data availability statement

The datasets presented in this article are not readily available per the determination of the Tulane University Institutional Review Board. Requests to access the datasets should be directed to kissing@tulane.edu.

## Ethics statement

The study involving human participants were reviewed and approved by Tulane University Institutional Review Board. Written informed consent from the participants' legal guardian/next of kin was not required to participate in this study in accordance with national legislation and the institutional requirements.

## Author contributions

JS drafted the manuscript, supported the conception, and performed data analysis and interpretation. AR and PK reviewed and edited the manuscript, supported the conception, and supported data analysis and interpretation. GG and PK developed the data collection instrument and GG oversaw data collection under the supervision of PK. HH supported the analytic methodology and reviewed data analysis. All authors contributed to the article and approved the submitted version.
